# Enabling the Triplet of Tetraphenylethene to Sensitize the Excited State of Europium(III) for Protein Detection and Time‐Resolved Luminescence Imaging

**DOI:** 10.1002/advs.201600146

**Published:** 2016-07-12

**Authors:** Zece Zhu, Bo Song, Jingli Yuan, Chuluo Yang

**Affiliations:** ^1^ Hubei Collaborative Innovation Center for Advanced Organic Chemical MaterialsHubei Key Lab on Organic and Polymeric Optoelectronic MaterialsDepartment of ChemistryWuhan UniversityWuhan430072P. R. China; ^2^ State Key Laboratory of Fine ChemicalsSchool of ChemistryDalian University of TechnologyDalian116024P. R. China

**Keywords:** europium complex, luminescence probe, protein detection, time‐resolved imaging

## Abstract

A tetraphenylethene (TPE) group that exhibits aggregation‐induced emission is incorporated into the ligand of a Eu(III) complex (TPEEu) to sensitize the excited state of Eu(III). In steady‐state measurements, TPEEu exhibits weak luminescence when dissolved in aqueous solutions even at a high concentration level, but emits strong fluorescence of TPE and phosphorescence of Eu(III) upon binding with bovine serum albumin. With a delay time of 0.05 ms and a gate time of 1.0 ms in time‐resolved measurements, only phosphorescent emission of Eu(III) is observed with a high on/off ratio. Moreover, this probe is successfully used in time‐resolved luminescence imaging to eliminate the background signal from biological autofluorescence without a washing process. This work provides a general strategy in designing Ln(III) complexes for detecting a broad range of biological molecules.

## 1 Introduction

Luminescence probes are widely used for biological assays and imaging in biochemical research and disease diagnosis.^1^ Because time‐resolved luminescence techniques operate within the time domain and are directed toward the detection of events that occur at much longer timescales (phosphorescence),^2^ various probes with long emission lifetimes (e.g., phosphorescence and delayed fluorescence) have been reported. With these probes, the time‐resolved techniques can eliminate the background signal from scattering and autofluorescence and greatly increase the signal‐to‐noise ratio.^3^ In contrast to other long‐lifetime luminescent probes that include transition‐metal complexes (e.g., Ru(II),^4^ Ir(III),^5^ and Pt(II)^6^) and delayed‐fluorescence compounds,^7^ Eu(III) and Tb(III) complexes possess luminescence lifetimes on the order of milliseconds; as such, Eu(III) and Tb(III) complexes can be distinguished more easily from other fluorophores possessing short fluorescence lifetimes in the nanosecond region.^8^ Because of their combined advantages of a large Stokes shift and sharp emission profile, Eu(III) and Tb(III) complexes are the most widely used luminophores in time‐resolved detection.^9^

However, Ln(III) ions exhibit weak absorption intensities that are difficult to be excited because the intraconfigurational f–f transitions are Laporte forbidden. This problem can be solved by incorporating a chromophore (i.e., a sensitizer or an antenna) into the ligand.^9^ The chromophore absorbs excitation light, converts to a triplet excited state by intersystem crossing and then transfers its energy to the Ln(III) ion. By correlating this sensitization process with target recognition, researchers have designed numerous luminescent Ln(III)‐complex‐based molecular probes for analytical and biological applications.^9^, ^10^, ^11^, ^12^, ^13^, ^14^, ^15^ Unfortunately, except for a few probes that exhibit significant luminescence enhancement from an initial low level upon binding^10^ or reacting^11^ with targets, many of the Ln(III) complex‐based probes exhibit self‐luminescence, which may result in high background signals.^1^, ^3^, ^12^, ^13^, ^14^, ^15^ In cell cultures, this problem may be avoided by washing the samples to remove unbound probes.^15^ However, in the case of living tissue or organism imaging, the in situ separation of unbound probes may be undesirable, difficult, or even impossible.^16^ In addition, this time‐consuming process delays the acquisition of microscopic data and prevents the monitoring of molecular interactions in real time.^17^ Therefore, it is highly demanding to design turn‐on luminescent probes with a high on/off ratio that may enable real‐time detection and achieve high‐contrast imaging. In this context, new strategies for sensitizing Ln(III) ions are to be developed.

**Scheme 1:**
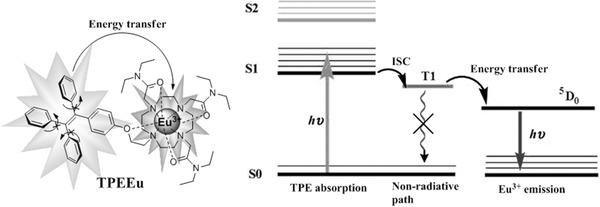
Structure of TPEEu (left) and schematic view of its sensitization processes (right).

In this article, we incorporated tetraphenylethene (TPE) in the ligand and designed a Eu(III) complex (TPEEu) probe (**Scheme** **1**). TPE‐based dyes are barely fluorescent in their separated monomer forms but highly emissive upon aggregation because of the restriction of intramolecular rotations.^18^, ^19^, ^20^ We expect that, when the intramolecular rotation of the TPE group is restricted, the nonradiative pathway of the TPE triplets is blocked and that the energy would transfer to the metal center (Scheme 1). This probe may exhibit a high on/off ratio because of the weak self‐luminescence due to nonradiative relaxations through intramolecular rotations. In addition, this probe can convert the blue fluorescence of TPE, which overlaps the emissions of biological autofluorescence, into the red phosphorescence of Eu(III) for time‐resolved detection.

## 2 Results and Discussion

### 2.1 Design and Synthesis of TPEEu

TPE‐based dyes have received much attention because of their relatively easy synthesis and because they are prone to further functionalization.^18^, ^19^, ^20^ Several TPE‐based fluorescent probes for the detection of bovine serum albumin (BSA) have been designed by Tang and co‐workers.^20^ These probes fluoresce upon binding to the BSA via noncovalent interactions, such as electrostatic and hydrophobic interactions. On the basis of the results of a previous study, we designed a new probe of TPEEu for the time‐resolved detection of BSA. We hypothesized that the TPE unit of TPEEu would bind to the hydrophobic pockets of BSA and then transfer its triplet energy to the Eu(III) ion. The synthetic route of TPEEu is shown in Scheme S1 (Supporting Information). Cross McMurry coupling of [4‐(2‐bromoethoxy)phenyl](phenyl) methanone with benzophenone yielded [2‐[4‐(2‐bromoethoxy)phenyl]ethene‐1,1,2‐triyl]tribenzene (**1**), which subsequently underwent two steps of substitution to afford ligand **3**. The reaction of **3** with the EuCl_3_ solution afforded the TPEEu complex (see the Supporting Information).

### 2.2 Spectroscopic Properties of TPEEu

**Figure 1:**
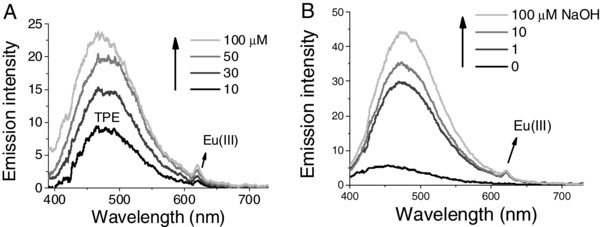
A) Steady‐state emission spectra of TPEEu in a 10 × 10^−3^ m HEPES (pH = 7) buffer. B) Steady‐state emission spectra of 50 × 10^−6^ m TPEEu upon addition of different concentrations of NaOH in water; *λ*_ex_ = 370 nm.

The steady‐state absorption (Figure S12, Supporting Information) and luminescence spectra (**Figure** **1**) of TPEEu in a HEPES (4‐(2‐hydroxyethyl)‐1‐piperazineethanesulfonic acid) buffer solution were measured. The complex exhibited an absorption peak at 300 nm (*ε*_max_ = 9420 m ^−1^ cm^−1^) and a main emission peak at about 460 nm, which originated from the TPE unit.^19^, ^20^ In addition, a very weak emission peak located at approximately 615 nm was considered to be the ^5^D_0_→^7^F_2_ transition of Eu(III),^1^, ^3^ which was due to the energy transfer from the TPE triplet to the metal center (Figure 1A). When the concentration of TPEEu was increased to 100 × 10^−6^ m in the solution, the luminescence assigned to the Eu(III) was still very weak (Figure 1A). The total luminescence quantum yield of this TPEEu solution turned out to be 0.07% by comparing with quinine sulfate in 0.1 m H_2_SO_4_ (*Φ* = 54%).^21^

The luminescence intensity of TPEEu was even weaker in pure water with a total luminescence quantum yield of 0.03% (Figure 1B). We speculated that this probe of TPEEu with its positive charges existed as a free monomer because of electrostatic repulsion with other probe molecules and that the excited energy was consumed through intramolecular rotations, leading to the weak luminescence. However, TPEEu showed more intensive luminescence in NaOH solutions (*Φ* = 0.23% in 0.1 × 10^−3^ m NaOH) (Figure 1B). We think the electrostatic repulsion was deceased in these alkaline solutions since OH^−^ can coordinate with Eu(III) and reduce its charges. The luminescence of TPEEu was also investigated in 90% glycerol‐in‐water as a viscous medium (Figure S14, Supporting Information). The emission intensity (*Φ* = 1.7%) was significantly stronger than that in water or buffer solution. Similar to many other fluorescent dyes that are sensitive to viscosity,^19^ the conformational restrictions can inhibit the nonradiative relaxations of the TPE excited state, leading to an increase in luminescence.

To study the energy transfer from the TPE unit to the metal center, the phosphorescence (T_1_→S_0_ transition) spectra of the ligand (compound **3**) in solid‐state at 77 K was measured (Figure S13, Supporting Information). The triplet state energy was estimated to be 2.45 eV (506 nm), which was higher than the ^5^D_0_ excited state of Eu(III) (2.14 eV, 580 nm).[[qv: 9a]] Besides, compound 3 exhibited stronger TPE emission than TPEEu in 90% glycerol‐in‐water (Figure S14, Supporting Information). This indicated the TPE unit could partly transfer its energy to metal center.

### 2.3 Detection of Proteins

**Figure 2:**
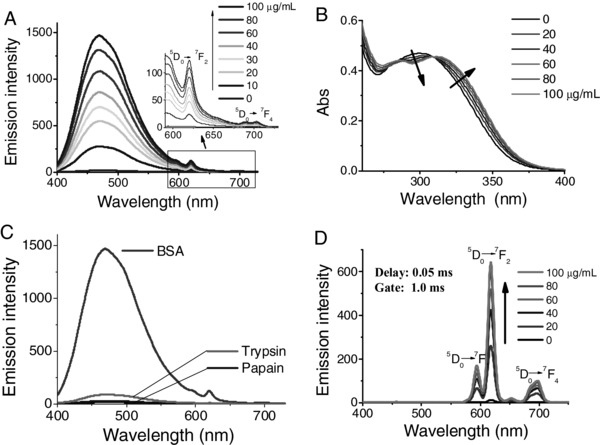
A) Steady‐state emission spectra of TPEEu upon addition of different concentrations of BSA. Inset: partially enlarged spectra. *λ*_ex_ = 370 nm. B) Absorption spectra of TPEEu upon addition of different concentrations of BSA. C) Steady‐state emission spectra of TPEEu in the presence of various proteins. [BSA] = [trypsin] = [papain] = 0.1 mg mL^−1^; *λ*_ex_ = 370 nm. D) Time‐resolved emission spectra of TPEEu upon addition of different concentrations of BSA; *λ*_ex_ = 330 nm. Buffer: 10 × 10^−3^ m HEPES. pH = 7. [TPEEu] = 50 × 10^−6^ m.

TPEEu was employed as a luminescent probe for protein detection. First, the steady‐state luminescence spectra during titration experiments with BSA were investigated (**Figure** **2**A). The TPE fluorescence (465 nm) of the probe increased significantly upon the addition of BSA. Simultaneously, the emission peaks located at 615 and 698 nm, which correspond to the ^5^D_0_→^7^F_2_ and ^5^D_0_→^7^F_4_ transitions of Eu(III), respectively, were also significantly enhanced. The emission intensities of the peaks located at 465 nm and 615 nm were approximately 93‐ and 48‐fold greater than their initial values, respectively, when the concentration of BSA reached 100 μg mL^−1^. The fluorescent and phosphorescent quantum yields of this solution turned out to be 3.5% and 0.06%, respectively, by comparison of the integrated areas of the corresponding emission peaks with that of quinine sulfate. Some other TPE probes exhibited slight red‐shift of the absorption upon binding to their target molecules, such as DNA and ATP.^23^ Similarly, in the absorption titration of TPEEu with BSA, a slight redshift was observed as the concentration of BSA increased (Figure 2B), which confirmed the binding of TPEEu with BSA.

We think the hydrophobic pockets of BSA may bind and restrict the TPE moieties of TPEEu. Therefore, the TPE triplet transferred its energy to Eu(III) and turned on the long‐lifetime phosphorescence emissions. The time‐gated excitation spectrum of TPEEu emitting at 616 nm in the presence of BSA overlapped well with the steady‐state excitation spectrum emitting at 460 nm (Figure S15, Supporting Information), which proves the antenna effect of the TPE unit as ligand. To evaluate the selectivity of the probe, trypsin and papain were also tested. TPEEu exhibited very weak luminescence in the presence of the two proteins (Figure 2C). This suggested the interactions between TPEEu and trypsin/papain were most likely very weak, or the intramolecular rotation of the TPE group was not effectively restricted. To distinguish the long‐lived luminescence from other emissions, time‐resolved spectra were measured under the same conditions (Figure 2D). With a delay time of 0.05 ms and a gate time of 1.0 ms, TPEEu exhibited clear phosphorescent emissions of Eu(III). The emission maximum located at ≈618 nm increased 47‐fold compared to its original value when the concentration of BSA reached 100 μg mL^−1^. This on/off ratio was much higher than that of many other time‐resolved probes based on Eu(III) or Tb(III) complexes.^12^, ^13^, ^14^, ^15^

### 2.4 Luminescence Decay of TPEEu

**Figure 3:**
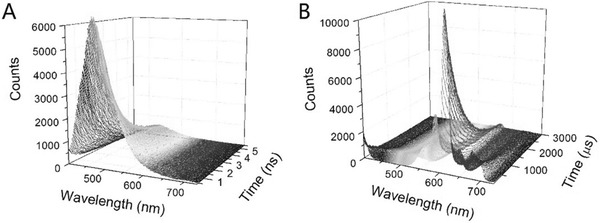
3D time‐resolved emission spectra of TPEEu in the presence of BSA in a 10 × 10^−3^ m HEPES buffer solution. [TPEEu] = 50 × 10^−6^ m. [BSA] = 100 μg mL^−1^. A) Excited by a pulsed diode laser (EPL‐375, wavelength: 377 nm). B) Excited by uF900; *λ*_ex_ = 330 nm.

Luminescence‐decay measurements were acquired using a transient‐state spectrometer. By recording the photon counts of each wavelength, we obtained the 3D time‐resolved emission spectrum of TPEEu in the presence of BSA (**Figure** **3**). The fluorescence of the TPE unit decayed quickly and was only observed in the nanosecond range (Figure 3A). However, the phosphorescence of Eu(III) lasted several milliseconds (Figure 3B). The lifetimes (*τ*) were obtained by fitting the luminescence‐decay data to a single‐exponential curve (**Table** **1**). In the absence of BSA, *τ*_615nm_ of TPEEu was ≈403 μs. Upon the addition of 100 μg mL^−1^ BSA, the lifetime increased to 856 μs.

**Table 1:** The luminescence lifetimes of TPEEu in different solutions. See Figures S16–S22 in the Supporting Information for fitting curves of the luminescence decay data and other details

	*τ*_460nm_ [ns]	*τ*_615nm_ [μs]
0.1 m NaOH in H_2_O	–	494
90% Glycerol in H_2_O	3.47	450
10 × 10^−3^ m HEPES in H_2_O	3.26	403
D_2_O	–	888
BSA in 10 × 10^−3^ m HEPES in H_2_O	3.22	856
BSA in D_2_O	3.00	1007

**Scheme 2:**
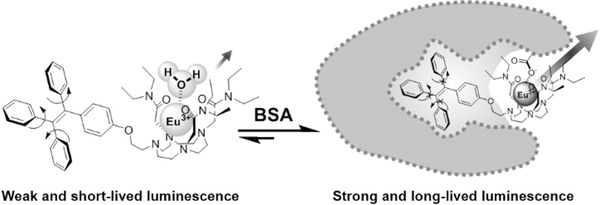
Proposed mode of TPEEu binding to BSA.

This change of lifetime can be explained by nonradiative deactivation processes upon interaction with OH oscillators from the solvent molecules.^1^ When TPEEu exists as a free monomer in solution, the water molecules coordinate with Eu(III) and quench its excited state, leading to a faster luminescence‐decay rate (**Scheme** **2**). To confirm this, the number of water molecules coordinated to the Eu(III) ion, *q*, was determined by using the method developed by Horrocks and co‐workers.^22^ The Eu(III) emission of TPEEu in pure H_2_O was so weak that its luminescence‐decay was difficult to measure. Instead, the phosphorescence lifetime of TPEEu (*τ*_615nm_) in 0.1 m NaOH and 90% glycerol was about 494 and 450 μs respectively, which was close to that in the HEPES buffer in the absence of BSA. In D_2_O, *τ*_615nm_ of TPEEu was ≈888 μs. It was much longer than that in solutions containing H_2_O, suggesting that a water molecule was bound to the TPEEu (*q* ≈ 1) in the absence of BSA. Upon the addition of 100 μg mL^−1^ BSA prepared in D_2_O, *τ*_615nm_ increased to 1007 μs, which indicated a *q* value of 0 in the complex formed between TPEEu and BSA. This probably because the amino acid residues (e.g., carboxyl) coordinated with Eu(III) upon binding with BSA, and the hydrophobic pockets of BSA protected the Eu(III) coordination sphere from water. Therefore, the luminescence was enhanced and lengthened, which would favor an increase in the signal‐to‐noise ratio by extending the gate time.

### 2.5 Biological Imaging

**Figure 4:**
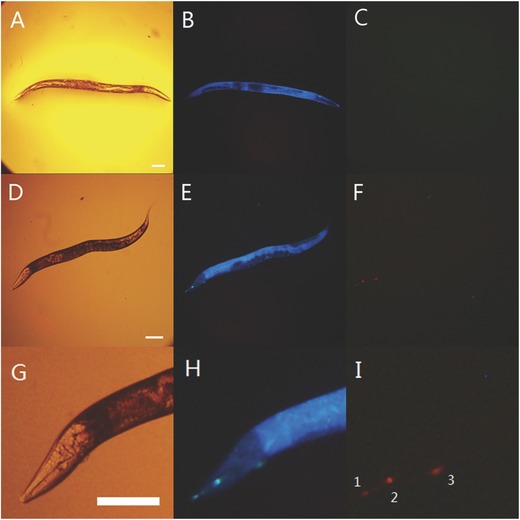
A) Bright‐field, B) steady‐state, and C) time‐resolved luminescence images of *C. elegans* in the absence of TPEEu. D) Bright‐field, E) steady‐state, and F) time‐resolved luminescence images of *C. elegans* incubated with 100 × 10^−6^ m TPEEu in water for 20 min. (G), (H) and (I) are partially enlarged images of (D), (E), and (F), respectively. The stained organs: 1: buccal cavity; 2: metacorpus; 3: excretory pore. Scale bar: 0.1 mm. Excitation filter, 330–380 nm.

The high luminescence signal‐to‐noise ratio suggested that TPEEu could be used in wash‐free in situ imaging of biological systems. Time‐resolved imaging was carried out by employing *Caenorhabditis elegans*, which is an important model system for biological research in many fields, including genomics, cell biology, and neuroscience.^24^ First, the autofluorescence of a wild‐type N2 worm was investigated in the absence of TPEEu (**Figures** **4**A–C). Notably, the pseudocoelom of the worm emitted blue autofluorescence in the steady‐state imaging mode (Figure 4B) and the time‐resolved luminescence image showed no long‐lived luminescence signals (Figure 4C). For comparison, another worm was incubated with 100 × 10^−6^ m TPEEu in water for 20 min and then subjected to microscopy imaging. The steady‐state luminescence image (Figures 4E,H) showed the blue autofluorescence from the pseudocoelom of the worm, similar to that in Figure 4B. In addition, some organs emitted bluish‐green luminescence, which was not observed in Figure 4B. Moreover, this emission changed to red luminescence, which was easily visible with a dark background in the time‐resolved imaging mode, with no autofluorescence (Figures 4F,I). This phenomenon may result from TPEEu being aggregated in these organs or restricted by the hydrophobic cavities of some molecules in these organs. In comparison to many other Eu(III) or Tb(III) complexes^12^, ^13^, ^14^, ^15^ used in time‐resolved microscopy imaging, this probe emitted weak luminescence when dissolved in solutions, even when dissolved at high concentrations. Therefore, this probe exhibits the potential for application in in situ biological imaging in cases where the excess probe molecules may be difficult to be washed out.

## 3 Conclusions

In conclusion, we incorporated the emission mechanism of restricting intramolecular rotations to design a new Eu(III) complex for time‐resolved assays and imaging. TPEEu exhibited weak luminescence when dissolved in aqueous solutions but emitted strongly upon binding with BSA. This probe exhibited a high on/off ratio, and was successfully used in time‐resolved imaging of *C. elegans* to eliminate the background signal of biological autofluorescence. All of these experiments confirmed that the tetraphenylethene unit of TPEEu can transfer its triplet energy to the metal center, and it is anticipated that this mechanism is very useful for the design of other Ln(III) complexes to detect a broad range of biological molecules.

## 4 Experimental Section

*Instrumentation*: UV–vis absorption spectra were recorded on a Shimadzu UV‐3600 recording spectrophotometer. Steady‐state emission spectra were collected on a Hitachi F‐4600 spectrophotometer. Time‐resolved emission spectra were recorded on a PerkinElmer LS‐55 spectrophotometer. 3D time‐resolved emission‐spectrum and luminescence decay curves were collected on an Edinburgh FLS920 spectrophotometer (see the Supporting Information for the parameter settings).

*Determination of Quantum Yield*: The quantum yields of fluorescence were determined by comparison of the integrated area of the emission spectrum of the samples with the reference of Quinine sulfate in 0.1 m H_2_SO_4_ (*Φ* = 54%).^21^ The quantum yields were calculated with the following expression:

$$\Phi_{x}=\Phi_{\mathrm{st}}\left(I_{x}/I_{\mathrm{st}}\right)\left(A_{\mathrm{st}}/A_{x}\right)$$

***Φ***_st_ is the reported quantum yield of the standard, *I* is the area under the emission spectra, ***A*** is the absorbance at the excitation wavelength 370 nm. All the fluorescence spectra were measured on a Hitachi F‐4600 spectrophotometer with same setting at room temperature.

*Imaging of C. elegans*: All bright‐field and luminescence imaging measurements were carried out on a laboratory‐use true color time‐gated luminescence microscope developed by Jin et al.[[qv: 2b]] For time‐resolved imaging measurements: Lamp pulse width: 80 μs; Excitation filter, 330–380 nm. Delay time: 33 μs; Gate time, 1.0 ms; Exposure time, 7 s.

*C. elegans* (N2) strains were cultured on nematode growth media (NGM) plates at 20 °C using OP50 *Escherichia coli* bacteria as food resources according to a previous protcol.[[qv: 24e,f]] To investigate the autofluorescence of *C. elegans*, a few worms were placed in a mixture of 100 μL of distilled water and 20 μL of ethanol and then viewed on a time‐gated luminescence microscope. For the control experiment, a few worms were placed in 100 μL of distilled water. A 1 μL solution of 10 × 10^−3^ m TPEEu was added to the water, and the worms were incubated for 20 min. Twenty microliters of ethanol was then added to this solution to stop the worms from wriggling, and a time‐gated luminescence microscope (objective, ×20; eyepiece, ×10) was subsequently used to view the worms.

## Supporting information

As a service to our authors and readers, this journal provides supporting information supplied by the authors. Such materials are peer reviewed and may be re‐organized for online delivery, but are not copy‐edited or typeset. Technical support issues arising from supporting information (other than missing files) should be addressed to the authors.

Supplementary
