# Supplementary material for: Enabling the Triplet of Tetraphenylethene to Sensitize the Excited State of Europium(III) for Protein Detection and Time‐Resolved Luminescence Imaging
Source: Adv Sci (Weinh). 2016 Jul 12;3(12):1600146. doi: 10.1002/advs.201600146 (PMC5157173; doi:10.1002/advs.201600146)
Supplement: Supplementary file 1 — Supplementary [file ADVS-3-0-s001.pdf]

Copyright WILEY-VCH Verlag GmbH & Co. KGaA, 69469 Weinheim, Germany, 2016.

ADVANCED SCIENCE
Open Access

Supporting Information

for *Adv. Sci.,* DOI: 10.1002/advs.201600146

Enabling the Triplet of Tetraphenylethene to Sensitize the Excited State of Europium(III) for Protein Detection and Time-Resolved Luminescence Imaging

*Zece Zhu, Bo Song, Jingli Yuan, and Chuluo Yang**

Supporting Information

Enabling the triplet of tetraphenylethene to sensitize the excited state of europium(III) for protein detection and time-resolved luminescence imaging

Zece Zhu,[1] Bo Song,[2] Jingli Yuan[1] and Chuluo Yang[*,1]

[1]Hubei Collaborative Innovation Center for Advanced Organic Chemical Materials, Hubei Key Lab on Organic and Polymeric Optoelectronic Materials, Department of Chemistry, Wuhan University, Wuhan, 430072, People's Republic of China.

[2]State Key Laboratory of Fine Chemicals, School of Chemistry, Dalian University of Technology, Dalian 116024, China

Table of Contents

1. Synthesis and characterisation......1
2. Spectra measurements......3
3. Reference......3
4. Figures......4

1. Synthesis and characterisation

Instrumentation

^{1}H NMR and ^{13}C NMR spectra were collected on a MECUYRVX300 spectrometer. Elemental analyses of carbon, hydrogen, and nitrogen were performed on a Vario EL III microanalyzer. EI-MS spectra were measured on a ThermoDSQ II 2000 spectrophotometer. ESI-MS spectra were measured on a Waters Micromass ZQ mass spectrometer.

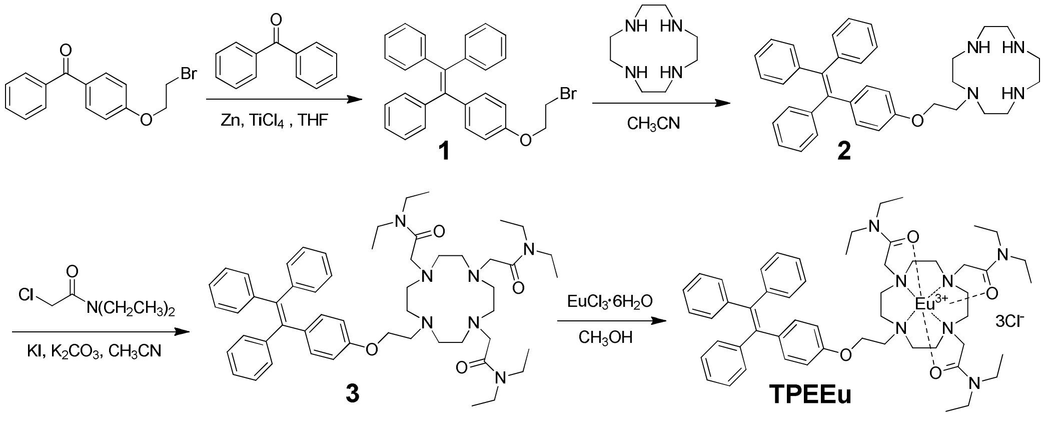

Scheme S1. The synthetic route of TPEEu

[4-(2-bromoethoxy)phenyl](phenyl)methanone was synthesized according to the reported literature.[1] All the other reagents were commercially available and used without further purification.

Synthesis of compound 1:

$TiCl_4$ (2 ml, 16 mmol) was added to a mixture of benzophenone (1.45 g, 8 mmol), [4-(2-bromoethoxy)phenyl](phenyl)methanone (2.45 g, 8 mmol) and Zn dust (2.14 g, 32 mmol) in anhydrous THF (180 mL) under argon with an ice-water bath. After refluxing for 21 hours, a suspension of $NaHCO_3$ (6.67g) was added to the flask and the mixture was then stirred until the solution faded. After filtration and solvent evaporation, the crude product was purified by a silica gel column using a mixture of dichloromethane/petroleum ether as gradient eluent to give a white powder (1.93 g, yield: 38%). ^{1}H NMR (300 MHz, $CDCl_3$), δ (ppm): 7.20-7.00 (m, 15H), 6.94 (d, *J* = 7.5 Hz, 2H), 6.64 (d, *J* = 6.9 Hz, 2H), 4.21 (t, *J* = 6 Hz, 2H), 3.60 (t, *J* = 6 Hz, 2H). ^{13}C NMR (75 MHz, $CDCl_3$), δ (ppm): 156.45, 143.76, 140.22, 136.77, 132.57, 131.25, 127.67, 127.56, 126.24, 113.71, 67.49, 29.11. EI-MS: *m/z* M^+: 454.3. Anal. Calcd for $C_{28}H_{23}BrO$: C, 73.85; H, 5.09; Found: C, 73.51; H, 4.81.

Synthesis of compound 2:

A mixture of 1,4,7,10-tetraazacyclododecane (0.69 g, 4 mmol) and compound **1** (0.46 g，1 mmol) in acetonitrile (40 ml) was refluxed under stirring for 48 h. After solvent evaporation, the crude product was purified by a silica gel column using methanol/aqueous ammonia (10:1, v/v) as eluent to give a white powder (0.39 g, yield: 71%). ^{1}H NMR (300 MHz, $CDCl_3$), δ (ppm): 7.15-6.95 (m, 15H), 6.89 (d, *J* = 9 Hz, 2H), 6.65 (d, *J* = 9 Hz, 2H), 3.96 (t, *J* = 5.7 Hz, 2H), 2.85 (t, *J* = 5.7 Hz, 2H), 2.77-2.61 (m, 4H), 2.68-2.61 (br, 8H), 2.55-2.48 (m, 4H). ^{13}C NMR (75 MHz, CD_3OD+CF_3COOD), δ (ppm): 161.27, 160.76, 160.25, 157.99, 145.31, 145.04, 141.87, 141.73, 138.32, 133.76, 132.27, 128.75, 128.66, 127.46, 127.35, 122.73, 115.09, 114.75, 95.38, 65.62, 52.13, 49.44, 45.60, 43.55, 43.07. ESI-MS: *m/z* $[M+H]^+$: 547. Anal. Calcd for $C_{36}H_{42}N_4O$: C, 79.08; H, 7.74; N, 10.25; Found: C, 78.94; H, 7.58; N, 10.07.

Synthesis of compound 3:

A mixture of compound **2** (0.08 g，0.146 mmol), *N,N*-diethylchloroacetamide (0.11 g, 0.74 mmol), potassium iodide (0.20 g, 1.2 mmol) and potassium carbonate (0.22 g, 1.6 mmol) in acetonitrile (10 ml) was refluxed under stirring for 48 h. After solvent evaporation, the crude product was purified by a silica gel column using chloroform/methanol (20:1, v/v) as eluent to give a colorless viscous liquid (0.12 g, yield: 93%). ^{1}H NMR (300 MHz, $CDCl_3$), δ (ppm): 7.20-6.90 (m, 15H), 6.86 (d, *J* = 8.7 Hz, 2H), 6.65 (d, *J* = 8.7 Hz, 2H), 4.35 (br, 2H), 4.08 (br, 2H), 3.40-3.10 (m, 18H), 2.95-2.30 (m, 16H), 1.22-0.9 (m, 18H). ^{13}C NMR (75 MHz, $CDCl_3$), δ (ppm): 169.52, 169.30, 157.13, 143.79, 140.21, 139.86, 135.97, 131.97, 127.55, 127.38, 126.18, 126.06, 113.98, 65.70, 54.82, 52.33, 50.47, 46.72, 41.08, 40.96, 40.33, 29.51, 14.28, 12.73, 12.59, 9.12. ESI-MS: *m/z* $[M+H]^+$: 886, $[M+Na]^+$: 908.

Synthesis of TPEEu:

A mixture of compound **3** (0.048 g, 0.053 mmol) and $EuCl_3$•$6H_2O$ (0.022 g, 0.06 mmol) in methanol (2.5 ml) was refluxed under stirring for 15 h. After filtration and solvent evaporation, the crude product was dissolved in 1ml methanol. 10 ml of ether was added to produce the precipitate. The precipitate was collected by filtration, washed with ether, and finally dried overnight in vacuum to give a yellow powder (53 mg, yield: 85%). See Figure S7 for ^{1}H-NMR.

^{13}C NMR (75 MHz, CD_3OD), δ (ppm): 158.93, 147.44, 145.47, 145.33, 145.29, 142.04, 141.88, 138.15, 134.02, 132.47, 132.21, 129.05, 128.87, 127.67, 127.56, 115.55, 115.52, 68.95, 66.94, 51.67, 38.90, 36.77, 26.57, 15.61, 14.73, 14.67, 12.19, 10.10. Anal. Calcd for $C_{54}H_{75}N_7O_4EuCl_3$ •$8H_2O$: C, 50.33; H, 7.12; N, 7.61; Found: C, 49.91; H, 7.57; N, 7.76.

2. Spectra measurements

Subsequent fluorescence titration experiments were carried out at room temperature by addition of proteins dissolved in distilled water into the buffer solutions. All the solutions were mixed fully and then measured on spectrophotometers.

UV–vis absorption spectra were recorded on a Shimadzu UV-3600 recording spectrophotometer.

Steady-state emission spectra were measured on a Hitachi F-4600 spectrophotometer. Parameters settings: EX Slit: 5.0 nm; EM Slit: 5.0 nm; Scan speed: 1200 nm/min; PMT Voltage= 700 V; Response= 0.5 s;

The emission spectra of compound **3** in solid-state at 77K were measured on a Hitachi F-4600 spectrophotometer.

Time-resolved emission spectra were measured on a PerkinElmer LS-55 spectrophotometer. Parameters settings: Delay: 0.05 ms; Gate: 1 ms; Slit: ex 10 nm; em 10 nm; Flash count: 1; Scan speed: 1200 nm/min;

3D time-resolved emission spectrum and luminescent decay curves were measured on an Edinburgh FLS920 spectrophotometer.

For 3D time-resolved emission spectrum measurements:

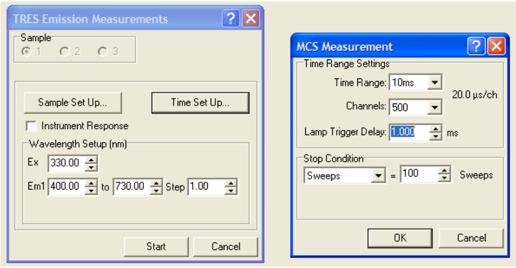

For luminescent measurements in the nanosecond region, a picosecond pulsed diode laser (EPL-375, wavelength: 377nm; pulse width: 79.6 ps) was used as lamp source. Parameters settings: Time calibration: 0.09766 ns; Channel range: 0 to 1023; Repeats: 1; Acq time: 2.1 s; Temperature: 28.4 °C; TCSPC mode Lifetime Parameters: TAC: 0.0ns, Delay: 0.0; Emission Arm Parameters: Slit size: 18.00 mm.

For luminescent measurements in the millisecond region: Parameters settings: Lamp properties: Lamp source: uF900, Frequency: 50.0 kHz; Slit size: Excitation: 2.00 mm; Emission: 18.00 mm.

3. Reference

(1) L. Xu, Z. Zhu, X. Zhou, J. Qin, C. Yang, *Chem. Commun.* ***2014***, **50**, 6494−6497.

4. Figures

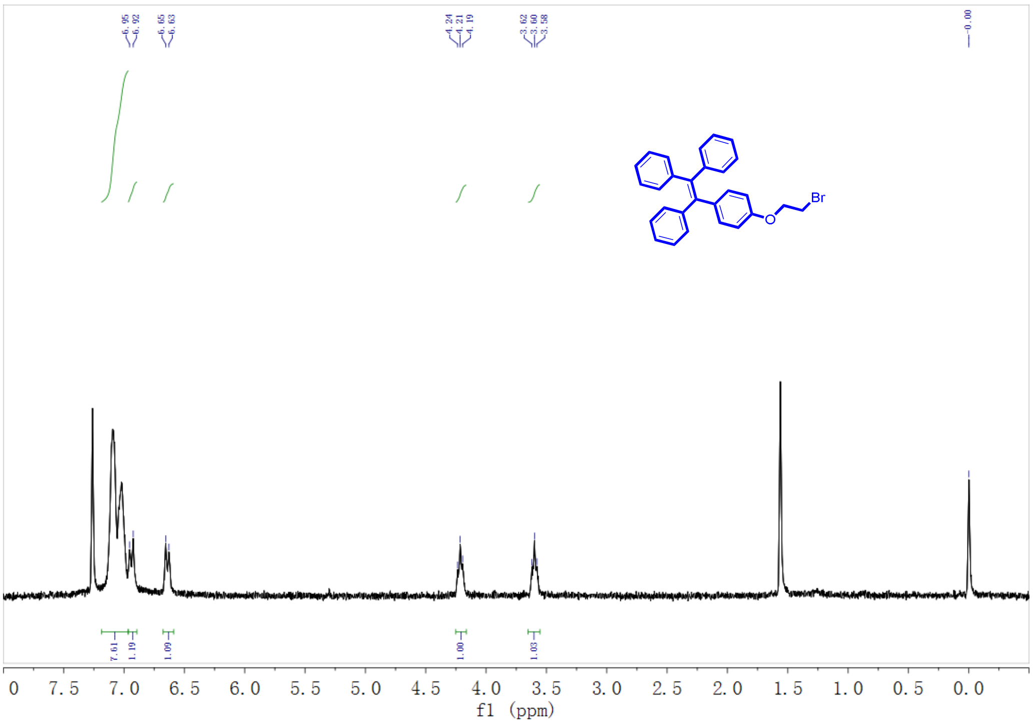

Figure S1. ^{1}H-NMR spectrum of compound **1** in $CDCl_3$.

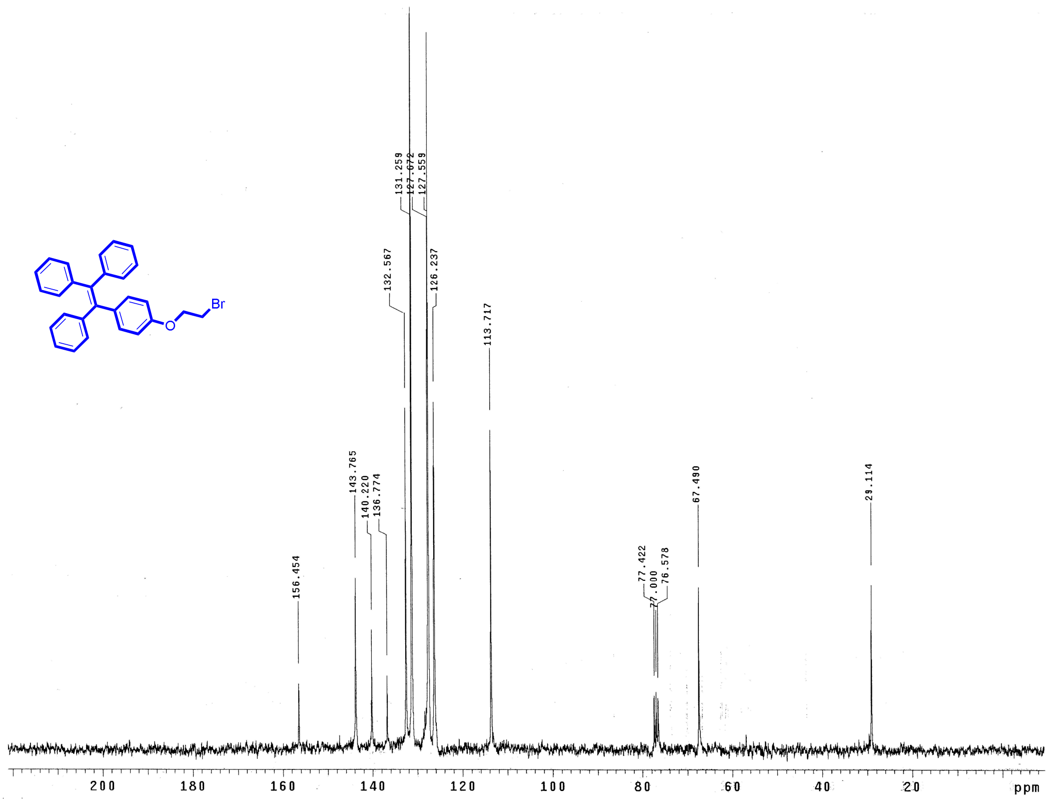

Figure S2. ^{13}C-NMR spectra of compound **1** in $CDCl_3$.

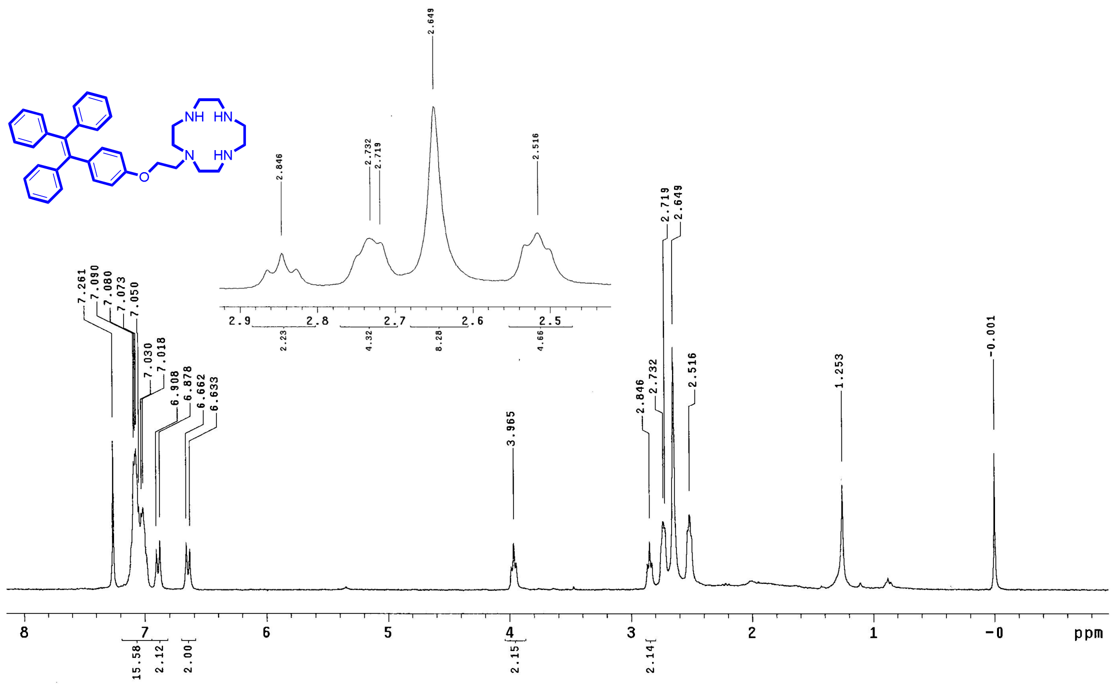

Figure S3. ^{1}H-NMR spectrum of compound **2** in $CDCl_3$.

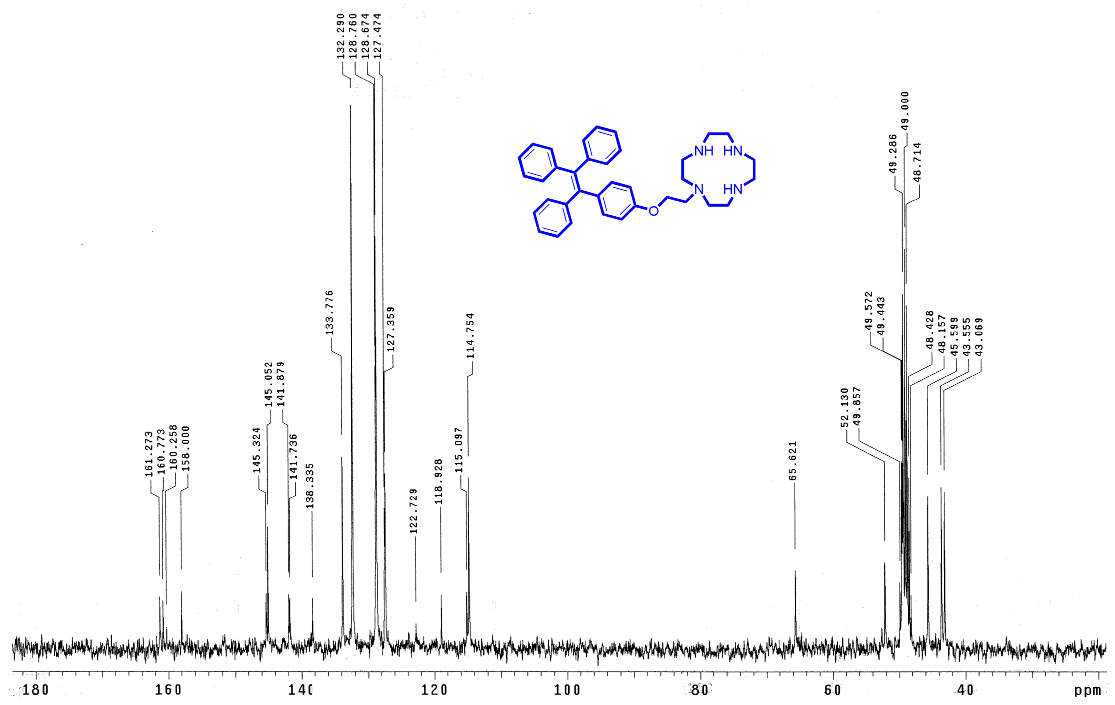

Figure S4. ^{13}C-NMR spectra of compound **2** in CD_3OD+CF_3COOD.

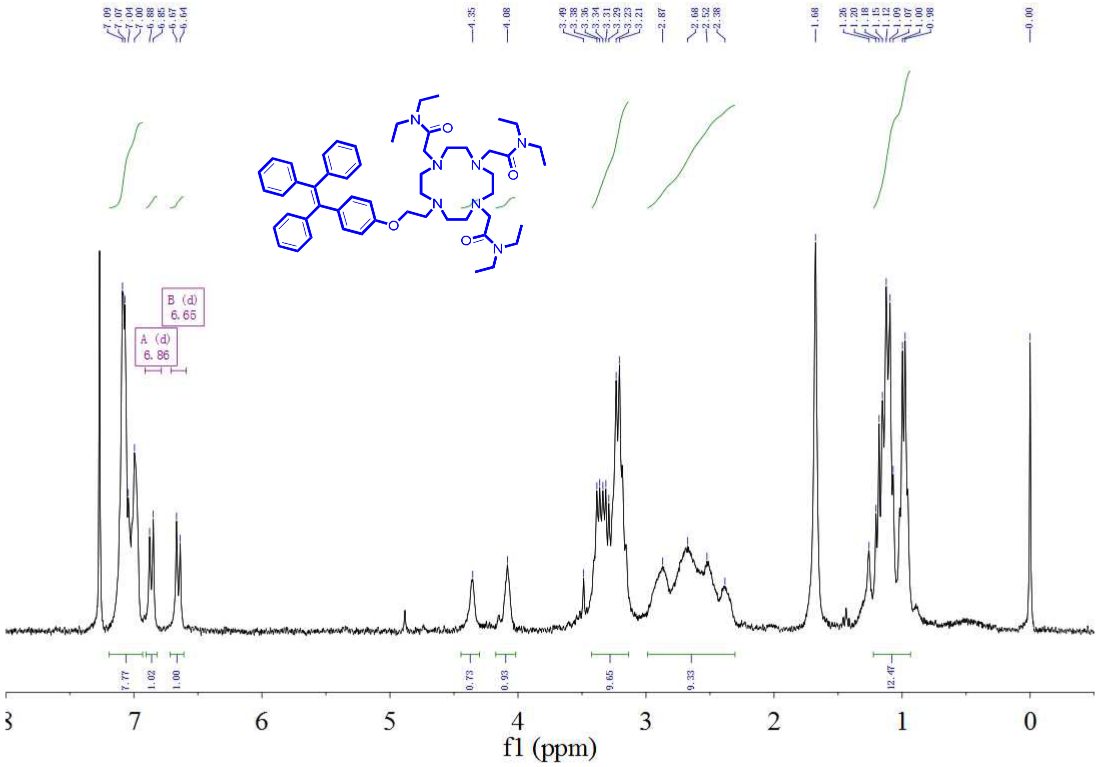

Figure S5. ^{1}H-NMR spectrum of compound **3** in $CDCl_3$.

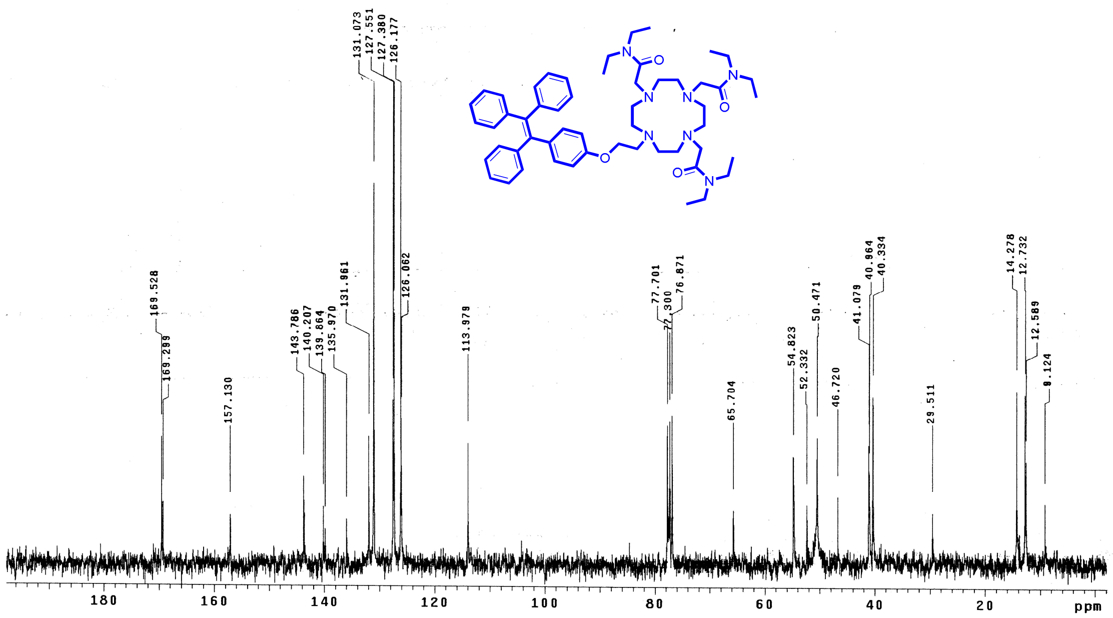

Figure S6. ^{13}C-NMR spectra of compound **3** in $CDCl_3$.

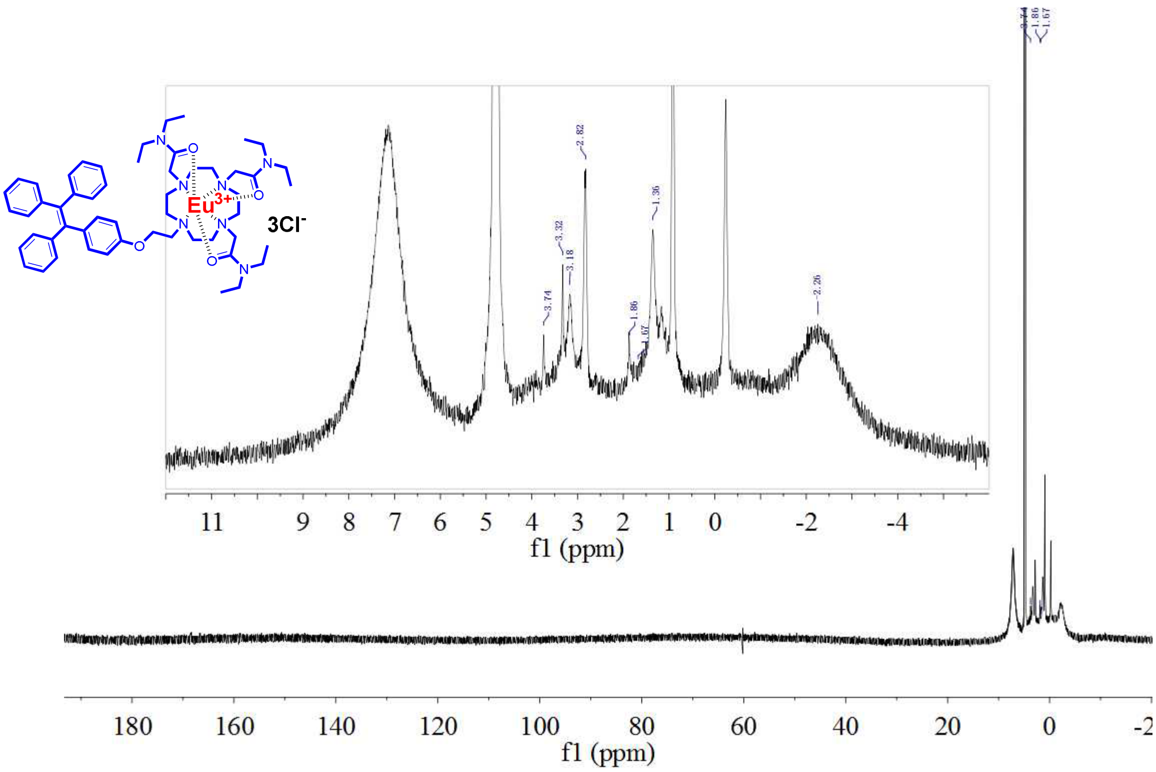

Figure S7. ^{1}H-NMR spectrum of TPEEu in D_2O.

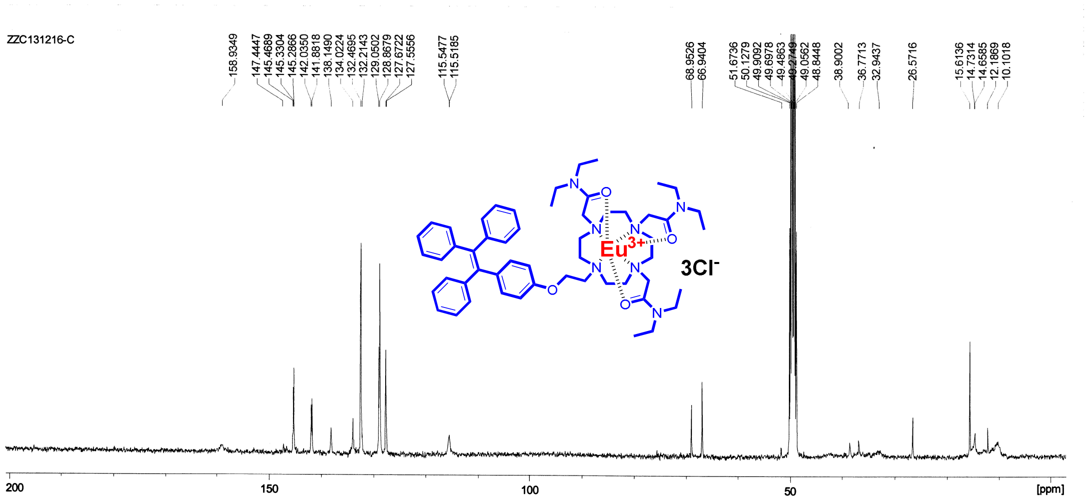

Figure S8. ^{13}C-NMR spectra of TPEEu in CD_3OD.

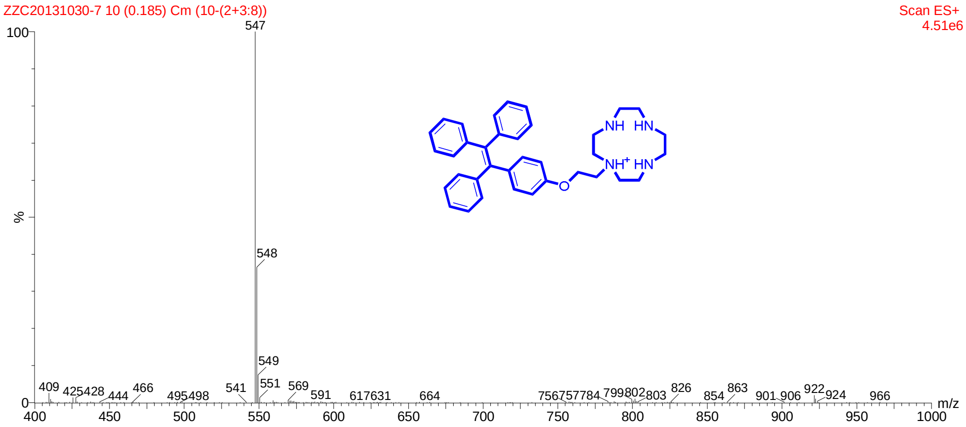

Figure S9. ESI-MS of compound **2** in methanol.

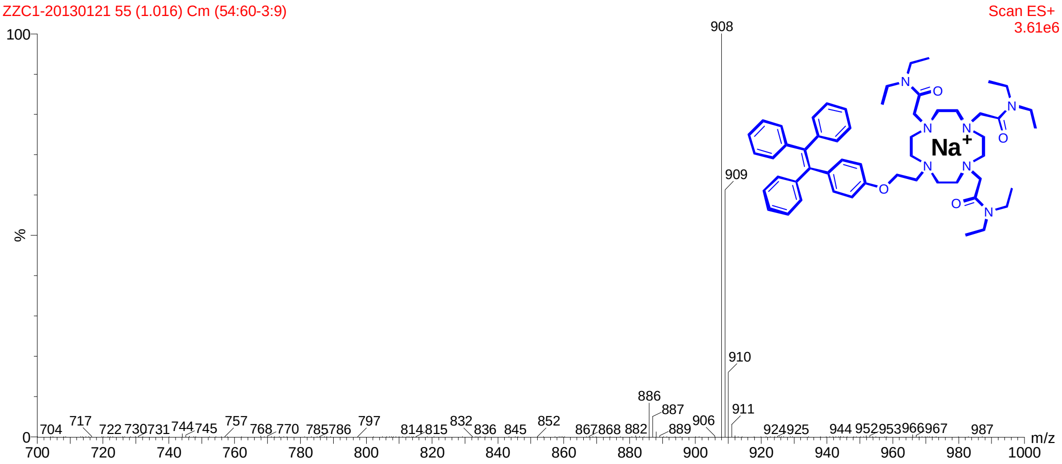

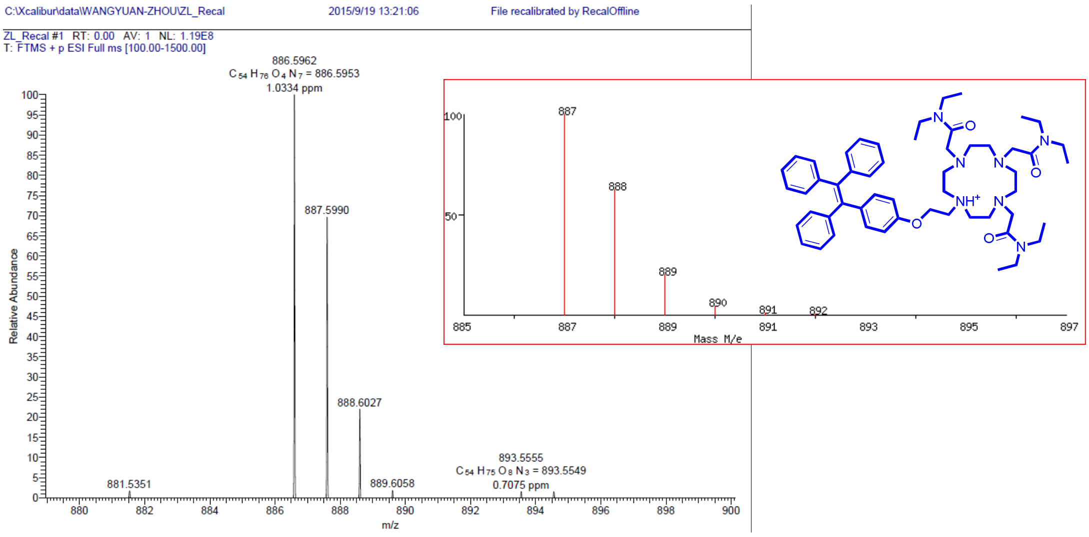

Figure S10. ESI-MS (up) and High resolution MS (down) of compound **3** in methanol. Insert: calculated distribution plot.

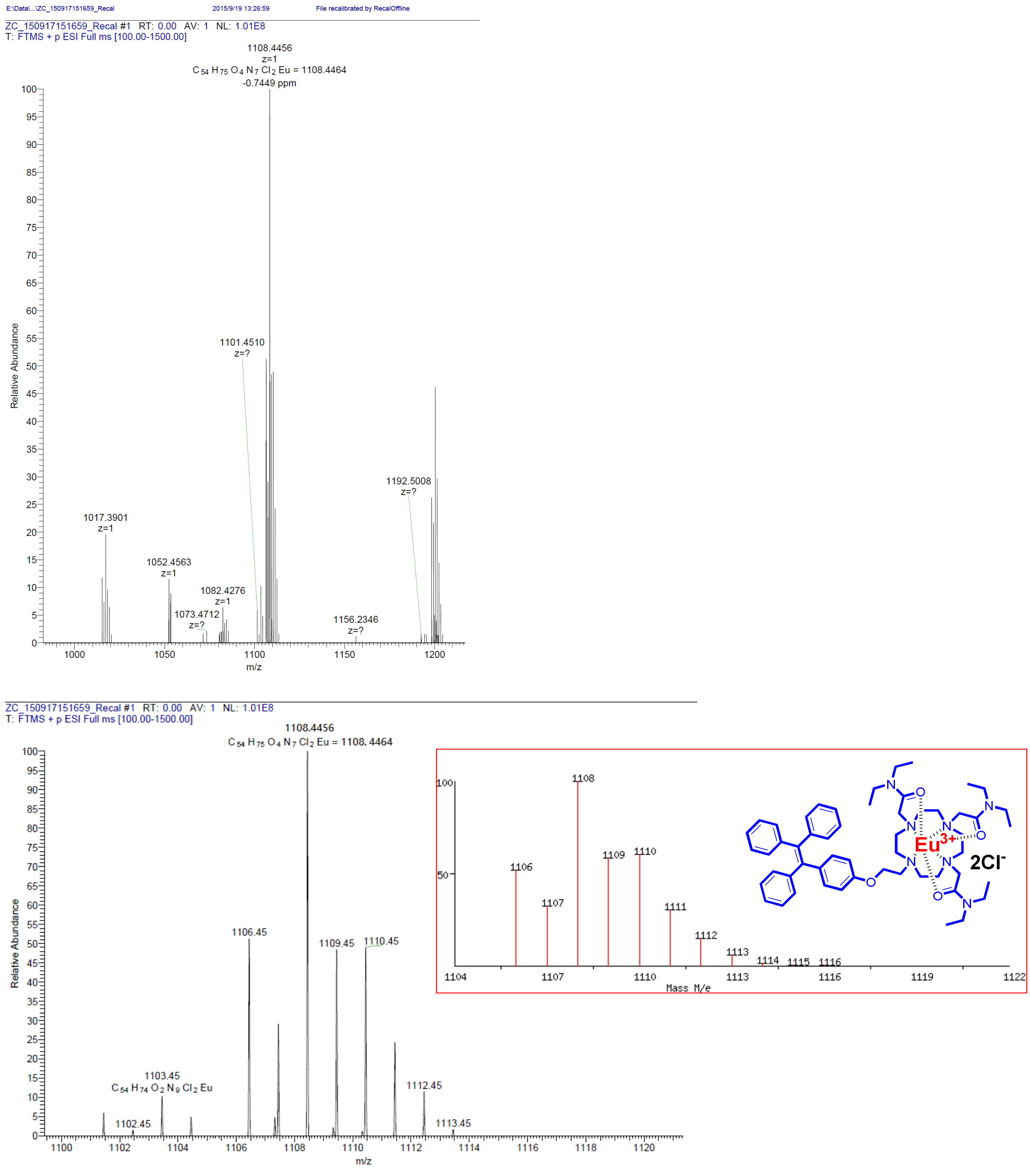

Figure S11. High resolution MS of TPEEu in methanol. Insert: calculated distribution plot.

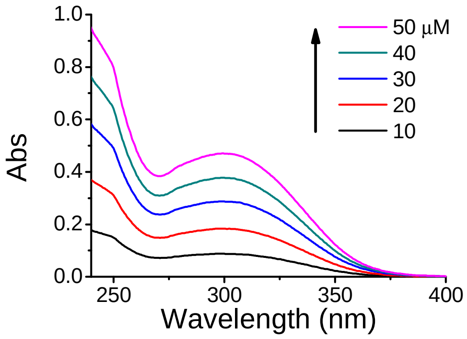

Figure S12. Absorption spectra of TPEEu in a 10 mM HEPES buffer solution. pH = 7.

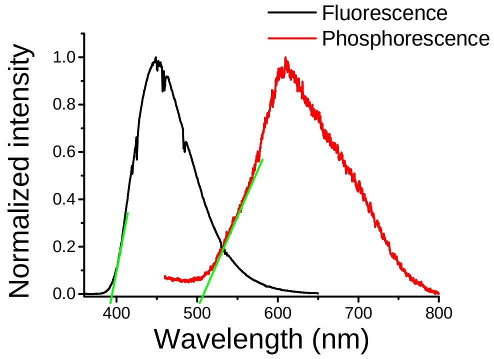

Figure S13. The emission spectra of compound **3** (ligand) in solid-state at 77K. The single state and triplet state energy were estimated to be 3.15 (394 nm) and 2.45 eV (506 nm) respectively.

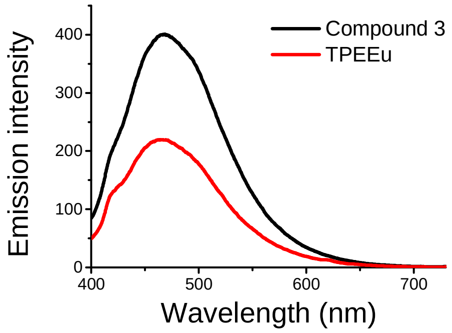

Figure S14. Steady-state emission spectra of compound **3** (ligand) and TPEEu in 90% glycerol-in-water. λ_{ex} = 370 nm. [compound **3**] = [TPEEu] = 50 μM.

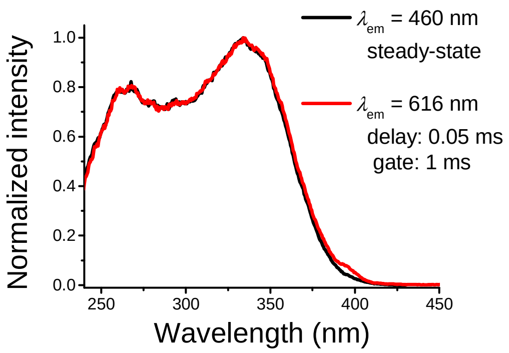

Figure S15. The steady-state and time-gated excitation spectra of the TPEEu in the presence of BSA in10 mM HEPES buffer solution. pH = 7. [TPEEu] = 50 μM. [BSA] = 100 μg/mL. Measured on LS-55.

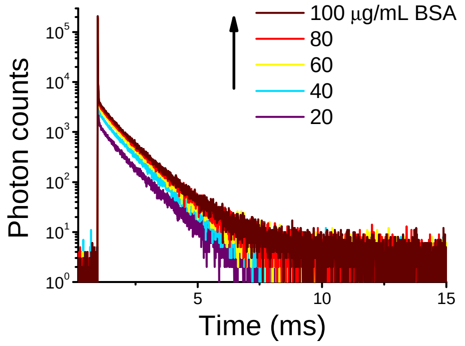

Figure S16. Luminescence decay of TPEEu in the presence of various amounts of BSA in a 10 mM HEPES buffer solution. [TPEEu] = 50 μM. λ_{ex} = 370 nm. λ_{em} = 615 nm.

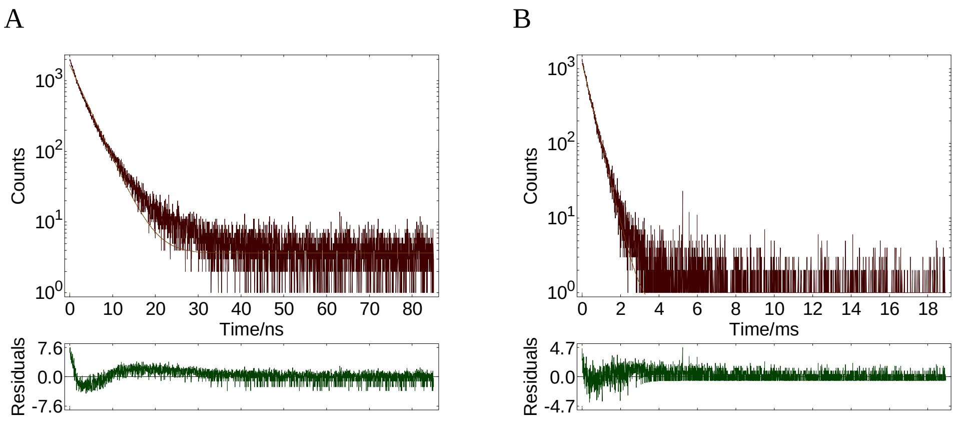

Figure S17. Single-exponential fitting of the luminescence decay of TPEEu in 10mM HEPES buffer. [TPEEu] = 500 μM. (A) λ_{ex} = 377 nm. λ_{ex} = 460 nm. τ = 3.26 ns, χ^2 = 2.277. (B) λ_{ex} = 330 nm. λ_{ex} = 615 nm. τ = 404 μs, χ^2 = 0.806.

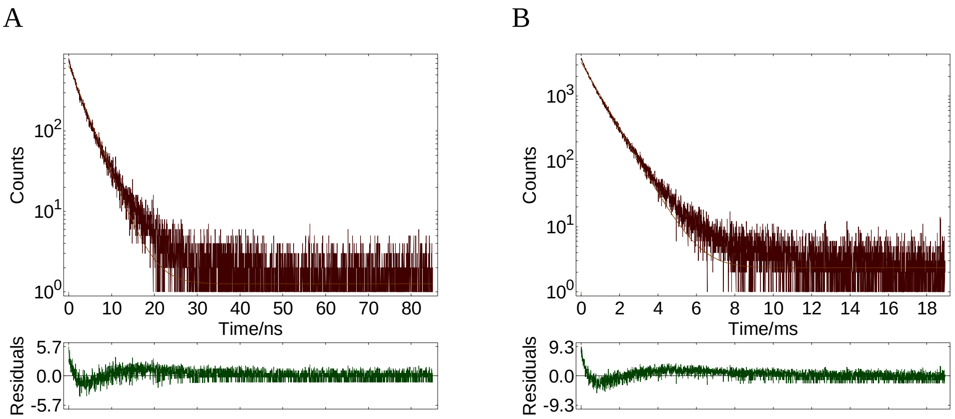

Figure S18. Single-exponential fitting of the luminescence decay of TPEEu in 10mM HEPES buffer with BSA. [TPEEu] = 50 μM. [BSA] = 100 μg/mL. (A) λ_{ex} = 377 nm. λ_{ex} = 460 nm. τ = 3.22 ns, χ^2 = 1.120. (B) λ_{ex} = 330 nm. λ_{ex} = 615 nm. τ = 856 μs, χ^2 = 2.467.

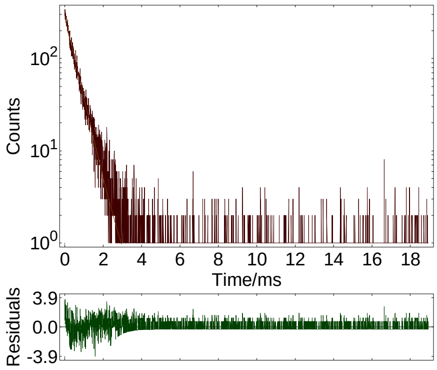

Figure S19. Single-exponential fitting of the luminescence decay of TPEEu in 0.1 mM NaOH. [TPEEu] = 50 μM. λ_{ex} = 330 nm. λ_{ex} = 615 nm. τ = 494 μs, χ^2 = 0.491.

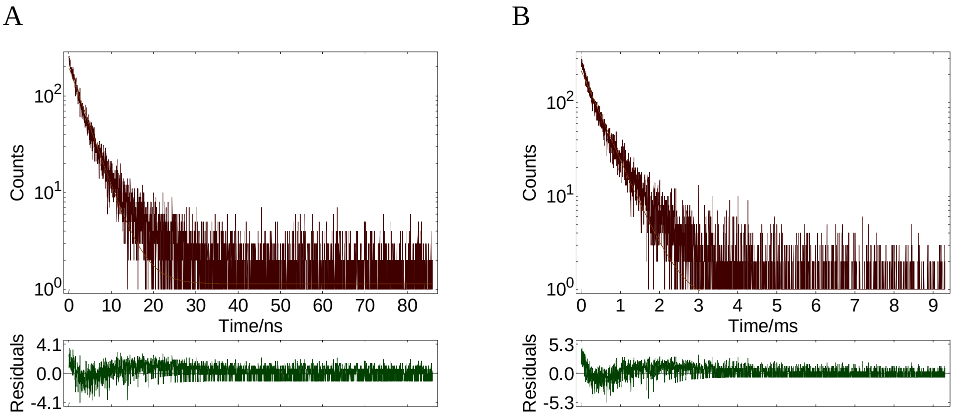

Figure S20. Single-exponential fitting of the luminescence decay of TPEEu in 90% glycerol. [TPEEu] = 50 μM. (A) λ_{ex} = 377 nm. λ_{ex} = 460 nm. τ = 3.47 ns, χ^2 = 0.995. (B) λ_{ex} = 330 nm. λ_{ex} = 615 nm. τ = 450 μs, χ^2 = 1.060.

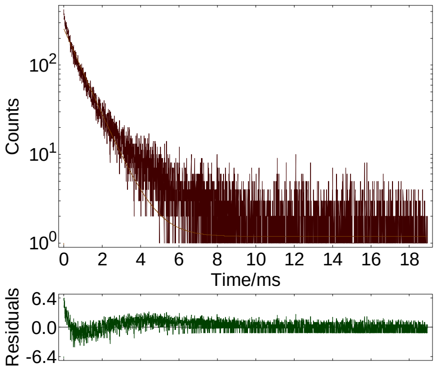

Figure S21. Single-exponential fitting of the luminescence decay of TPEEu in D_2O. [TPEEu] = 50 μM. λ_{ex} = 330 nm. λ_{ex} = 615 nm. τ = 888 μs, χ^2 = 1.490.

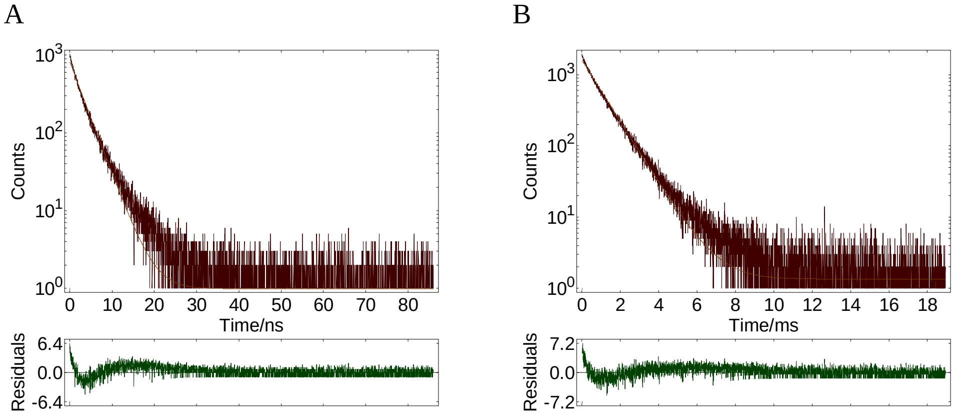

Figure S22. Single-exponential fitting of the luminescence decay of TPEEu in D_2O with BSA. [TPEEu] = 50 μM. [BSA] = 100 μg/mL. (A) λ_{ex} = 377 nm. λ_{ex} = 460 nm. τ = 3.00 ns, χ^2 = 1.259. (B) λ_{ex} = 330 nm. λ_{ex} = 615 nm. τ = 1007 μs, χ^2 = 1.631.

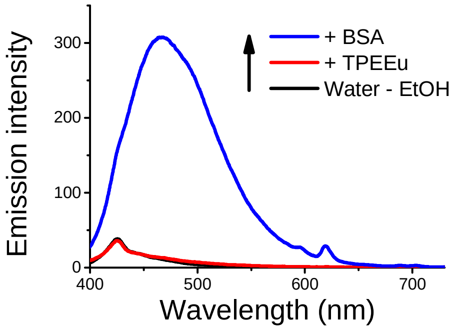

Figure S23. Steady-state emission spectra of TPEEu upon addition of BSA in a mixture of water and ethanol (v:v = 5:1). λ_{ex} = 370 nm. Buffer: 10 mM HEPES. pH = 7. [TPEEu] = 50 μM. [BSA] = 0.1 mg/mL.
